# Nitrous Oxide Emissions from Nitrite Are Highly Dependent on Nitrate Reductase in the Microalga *Chlamydomonas reinhardtii*

**DOI:** 10.3390/ijms23169412

**Published:** 2022-08-20

**Authors:** Carmen M. Bellido-Pedraza, Victoria Calatrava, Angel Llamas, Emilio Fernandez, Emanuel Sanz-Luque, Aurora Galvan

**Affiliations:** 1 Department of Biochemistry and Molecular Biology, University of Cordoba, 14004 Cordoba, Spain; 2 Department of Plant Biology, Carnegie Institution for Science, Stanford, CA 94305, USA

**Keywords:** nitrous oxide emission, carbon dioxide emission, nitrate reductase, nitrite reductase mutants, nitric oxide, *Chlamydomonas*

## Abstract

Nitrous oxide (N_2_O) is a powerful greenhouse gas and an ozone-depleting compound whose synthesis and release have traditionally been ascribed to bacteria and fungi. Although plants and microalgae have been proposed as N_2_O producers in recent decades, the proteins involved in this process have been only recently unveiled. In the green microalga *Chlamydomonas reinhardtii*, flavodiiron proteins (FLVs) and cytochrome P450 (CYP55) are two nitric oxide (NO) reductases responsible for N_2_O synthesis in the chloroplast and mitochondria, respectively. However, the molecular mechanisms feeding these NO reductases are unknown. In this work, we use cavity ring-down spectroscopy to monitor N_2_O and CO_2_ in cultures of nitrite reductase mutants, which cannot grow on nitrate or nitrite and exhibit enhanced N_2_O emissions. We show that these mutants constitute a very useful tool to study the rates and kinetics of N_2_O release under different conditions and the metabolism of this greenhouse gas. Our results indicate that N_2_O production, which was higher in the light than in the dark, requires nitrate reductase as the major provider of NO as substrate. Finally, we show that the presence of nitrate reductase impacts CO_2_ emissions in both light and dark conditions, and we discuss the role of NO in the balance between CO_2_ fixation and release.

## 1. Introduction

Nitrous oxide (N_2_O) is a greenhouse gas ~300-fold more potent than CO_2_ and considered the dominant ozone-depleting chemical emitted in the 21st century [1,2,3,4,5]. In 2020, the atmospheric N_2_O reached 333.2 ppb, which constitutes 123% of the pre-industrial (before 1750) levels, with the fastest-growing rate occurring in the past five decades [6,7,8]. N_2_O emissions are released to the atmosphere from natural (~60%) and anthropogenic sources (~40%), including oceans, soils, biomass burning, fertilizers, and several industrial activities. N_2_O emissions derived from human activities are dominated by nitrogen additions to crop plants [6,8]. In modern agriculture, the abundant supply of nitrogen fertilizers leads to excess nitrogen in the soil, and non-assimilated nitrogen can be emitted as N_2_O to the atmosphere or lost as runoffs into aquatic ecosystems, causing their eutrophication [9,10]. Nitrification and denitrification are two well-documented biochemical processes that control N_2_O emissions in terrestrial and aquatic ecosystems and are regulated by biological and environmental factors [8,11].

Bacteria and fungi are widely recognized as N_2_O producers by the scientific community [1,11,12,13,14], but recently, plants and algae have also emerged as N_2_O emitters. In the late 1970s, Hahn and Junge already hypothesized that phytoplankton and plants could release N_2_O in the presence of nitrate (NO_3_^−^) and nitrite (NO_2_^−^) [15]. Several years later, this was demonstrated in microalgae [16,17] and plant leaves during photosynthesis [18,19,20,21,22]. Despite this, the intergovernmental agencies have not yet considered N_2_O emissions by plants and algae in the global budget [6,23]. Thus, understanding the molecular mechanisms associated with N_2_O synthesis and their regulation under different environmental conditions is critical to assessing the actual contribution of plants and microalgae to atmospheric N_2_O emissions.

The molecular players for N_2_O synthesis are just starting to be studied in microalgae. *Chlamydomonas reinhardtii* (hereafter *Chlamydomonas*) is a unicellular, biflagellate, and green alga widely used as a model organism due to the numerous tools available to perform genetic and metabolic studies and its suitability for biotechnological applications [24,25]. Recently, two works have identified the flavodiiron proteins (FLVs) and cytochrome P450 (CYP55) as NO reductases (NORs) in *Chlamydomonas* [26,27]. The *Cr*FLVs belong to a singular family of O_2_ and NO reductases that are ubiquitous in oxygenic photoautotrophs, including cyanobacteria, the rhizarian *Paulinella chromatophora*, green algae, mosses, lycophytes, and gymnosperms, but are absent in angiosperms [28,29]. The *Chlamydomonas* CYP55 is a cytochrome p450 NO reductase closely related to fungal p450 nor members, which are considered biomarkers for N_2_O production [14]. In *Chlamydomonas*, CYP55 and FLVs are proposed to be responsible for N_2_O production in dark and light conditions, respectively [26,27]. In addition, light and dark N_2_O emissions have been studied in different algal species and correlated with the presence of *FLV* and *CYP55* genes in their genomes; algal species having only *FLV* (*Tetraselmis subcordiformis* and *Coccomyxa subellipsoidea*) seem to synthesize N_2_O in the light but not in the dark. In contrast, algae lacking both *CYP55* and *FLV (Galdieria sulphuria*, *Pophyridium purpureum*, *Nannochloropsis gaditana*, *Phaeodactylum tricornutum*, and *Thalassiosira pseudonana*) do not reduce NO to N_2_O, whereas those algal species containing both genes in their genome (*Chlamydomonas reinhardtii* and *Chlorella variabilis*) exhibit N_2_O emissions in light and dark conditions [27].

Both FLV and CYP55 require NO as substrate in order to synthesize N_2_O. Several NO synthesis pathways have been proposed in photosynthetic organisms. The most characterized mechanism entails the reduction of NO_2_^−^ to NO in a process mediated by the cytosolic NO_3_^−^ reductase (NR) in microalgae and plants [17,30,31,32,33]. These NRs are typical eukaryotic and nitrogen assimilatory enzymes that form homodimers containing FAD, heme b_557,_ and molybdenum cofactor as prosthetic groups [34,35]. These cofactors allow the sequential electron transfer from NAD(P)H to the molybdenum cofactor, the final electron donor for NO_3_^−^ reduction. The formed NO_2_^−^ is assimilated in the chloroplast by the following actions of NO_2_^−^ reductase (NiR) and glutamine synthetase (GS) [36]. In *Chlamydomonas*, the NR-dependent NO synthesis requires the protein partner ARC (aka. NOFNiR), a molybdoenzyme that accepts electrons from the NR heme group to reduce NO_2_^−^ to NO [35,37]. Moreover, the *Chlamydomonas* NR can also donate electrons to the truncated hemoglobin THB1 to scavenge NO and produce NO_3_^−^ by deoxygenation [38,39]. Thus, NR has a central role comprising the recently named NO_3_^−^-NO_3_^−^ cycle [29].

In this work, we use previously isolated *Chlamydomonas* NO_2_^−^ reductase mutants, which cannot assimilate NO_2_^−^, as a valuable tool to study NO_2_^−^ dissimilation to N_2_O. We show that the NR–ARC complex strongly contributes to N_2_O emissions in cells incubated in the presence of NO_2_^−^. Our results corroborate NR function in synthesizing NO and suggest that this cytosolic enzyme is the primary NO source for N_2_O synthesis, carried out in the chloroplast and mitochondria. Furthermore, we show that excess NO_2_^−^ and NR-dependent NO impacts CO_2_ emissions under our experimental conditions, and we briefly discuss the impact on CO_2_ fixation and release.

## 2. Results

### 2.1. Nitrite Reductase Mutants (nii1) Cannot Use NO_3_^−^/NO_2_^−^ for Growth but Can Reduce Them to N_2_O

**Figure 1:**
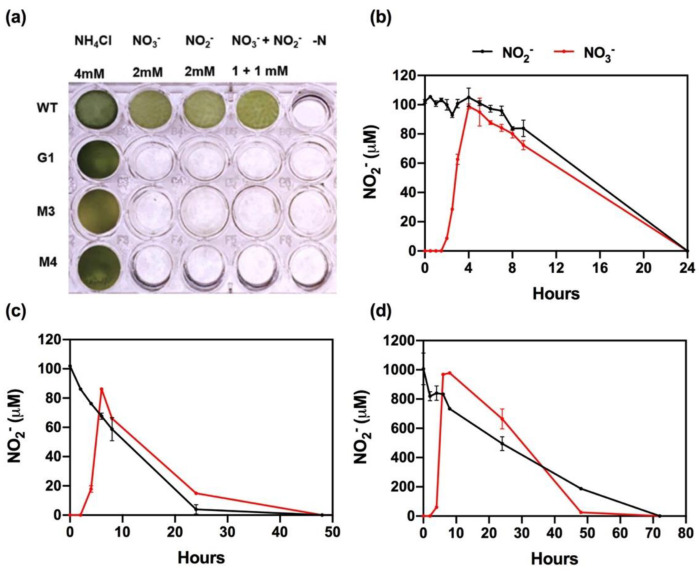
M3 strain cannot grow in NO_3_^−^ or NO_2_^−^ but metabolizes them. (**a**) Growth test of the *nii1* mutants (G1, M3, and M4) and the WT (6145c) strain on the indicated N sources. Plate wells were inoculated with 0.1 × 10^6^ cells ml^−1^ and cultured for 7 days. (**b**–**d**) Extracellular NO_2_^−^ quantification in cultures incubated in the presence of either NO_3_^−^ (red line) or NO_2_^−^ (black line). NH_4_^+^-grown cells were washed and transferred to NO_3_^−^- or NO_2_^−^-containing media at 0.1 mM in non-sealed bottles (**b**), 0.1 mM in sealed bottles (**c**), or 1 mM in sealed bottles (**d**). Error bars represent ±SD, *n* ≥ 3.

*Chlamydomonas nii1* mutants (G1, M3, and M4) cannot reduce NO_2_^−^ to ammonium (NH_4_^+^) and, therefore, do not grow in media containing either NO_3_^−^ or NO_2_^−^ as the sole nitrogen (N) source (Figure 1a). The G1 strain is a deletion mutant that lacks the entire cluster of the NO_3_^−^ assimilation genes. This cluster, located in chromosome 9, contains the genes that encode NO_3_^−^ and NO_2_^−^ reductases (*NIA1* and *NII1*, respectively) and the high-affinity NO_3_^−^/NO_2_^−^ transport components (*NRT2.1*, *NRT2.2*, and *NAR2*) [40,41]. By genetic crosses, either the *NIA1* and *NRT2.1*-*NAR2* sets of genes or only *NIA1* were transferred to the G1 strain, generating the M3 and M4 mutants, respectively (see [41] for more details). As previously mentioned, *Chlamydomonas* cells can reduce NO_2_^−^ to NO [37] and NO to N_2_O [26,27]; therefore, we used these mutants as model organisms to study this process in microalgae. First, we studied NO_2_^−^ evolution in the M3 strain. NH_4_^+^-grown cells were washed and transferred to fresh media containing 0.1 and 1 mM NO_3_^−^ or NO_2_^−^, and NO_2_^−^ concentration in the medium was determined at different time points (Figure 1b–d). Cells exposed to 0.1 mM NO_3_^−^ showed a stoichiometric excretion of NO_2_^−^ after 4 h (Figure 1b), as previously reported [41]. Subsequently, extracellular NO_2_^−^ concentration slowly decreased, being completely exhausted from the medium after 24 h. Similar depletion rates and kinetics were observed when 0.1 mM NO_2_^−^ was added instead, but a lag of 4–6 h was observed before the concentration started to decrease.

The same experiment was performed in sealed bottles, in which N_2_O emission would be retained and could be quantified. Under these conditions, similar rates of accumulation and depletion of NO_2_^−^ were observed (Figure 1c). However, NO_2_^−^ depletion was induced faster than in non-sealed cultures (2 h vs. 6–8 h); therefore, NO_2_^−^ excretion after NO_3_^−^ reduction was not stoichiometric and reached only a concentration of 86 µM. Furthermore, as observed in non-sealed bottles, NO_2_^−^ was exhausted before 24 h. A similar pattern was observed when cells were exposed to 1 mM NO_3_^−^ or NO_2_^−^, although total depletion required longer incubations (Figure 1d).

**Figure 2:**
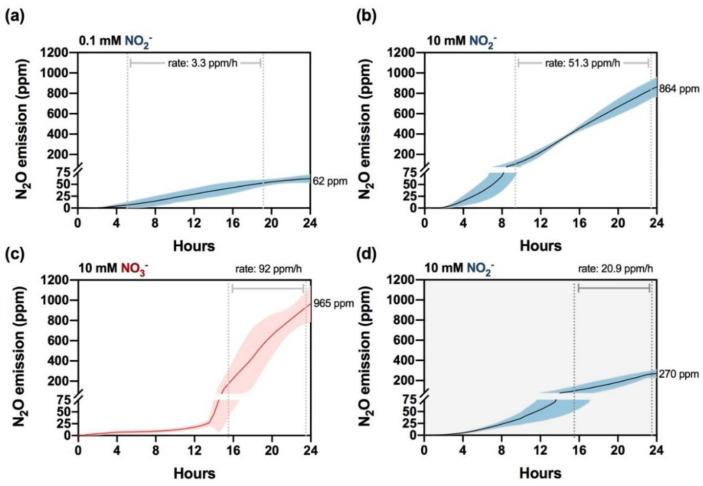
Kinetics of N_2_O emission in the M3 strain in the presence of NO_3_^−^ and NO_2_^−^. NH_4_^+^-grown cells were washed and transferred to NO_2_^−^-containing media in sealed bottles at 0.1 mM (**a**) and 10 mM (**b**) in the light. Cells were also incubated in the presence of 10 mM NO_3_^−^ under illumination (**c**) and 10 mM NO_2_^−^ in the dark (**d**). Each data line represents an average of three biological replicates, and the colored area corresponds to ±SD.

To monitor N_2_O emissions in the headspace of the cultures, we used Cavity Ring-Down Spectroscopy (CRDS) (see Material and Methods), which allows continuous N_2_O measurements. The M3 cultures produced N_2_O in a NO_2_^−^ concentration-dependent manner and from both NO_3_^−^ and NO_2_^−^ (Figure 2). When the cells were incubated with 0.1 mM NO_2_^−^, N_2_O started to accumulate after 2–3 h with a rate of 3.3 ppm/h and plateaued after 21 h, reaching a final concentration of 62 ppm after 24 h (Figure 2a). In the presence of 10 mM NO_2_^−^, although N_2_O accumulation was also detected after 2 h of induction, the gas was released at ~15-fold higher rate (51 ppm/h) than in 0.1 mM NO_2_^−^, and no saturation was observed after 24 h when N_2_O concentration was 864 ppm (Figure 2b). When the cells were incubated with 10 mM NO_3_^−^ (Figure 2c), N_2_O release was delayed as expected due to the requirement to reduce NO_3_^−^ to NO_2_^−^, but the production rate was boosted after 14 h (92 ppm/h), almost doubling that observed in the cells supplemented with NO_2_^−^. As expected, cells incubated in N-free media did not emit detectable amounts of N_2_O (Supplementary Table S1).

In *Chlamydomonas*, N_2_O production may involve light-dependent and light-independent pathways [26,27]; therefore, we additionally studied N_2_O production in cells incubated with NO_2_^−^ in the dark. In this condition, total N_2_O accumulation (270 ppm) and production rate (20 ppm/h) were both strongly reduced (Figure 2d), highlighting the importance of light in this process in the M3 strain.

In these experiments, the earliest N_2_O emissions were achieved during incubation with 10 mM NO_2_^−^, a concentration previously used by Plouviez and collaborators [26]; therefore, we set this concentration for further studies. Moreover, the kinetics and high rates of N_2_O production observed in the M3 strain led us to use this mutant as a model to study the role of other players involved in the reduction of NO_2_^−^ to N_2_O.

### 2.2. Nitrate Reductase Is the Primary NO Source Involved in N_2_O Emissions from NO_2_^−^ in the nii1 Mutants

**Figure 3:**
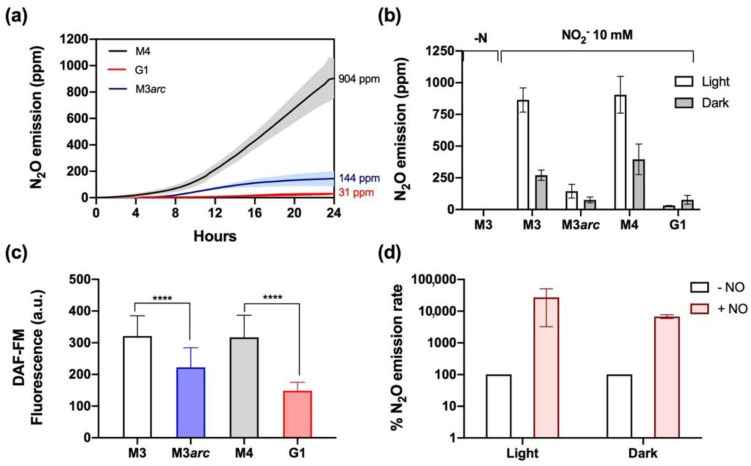
N_2_O emission by the *nii1* mutants mainly depends on the functionality of the NR–ARC complex to reduce NO_2_^−^ to NO. (**a**) N_2_O emissions by M3, G1, and M3*arc* strains in media containing 10 mM NO_2_^−^ in the light. (**b**) Effect of light and dark on total N_2_O emission in the *nii1* mutants in the presence of 10 mM NO_2_^−^ after 24 h. (**c**) DAF-FM fluorescence in the *nii1* mutants after 24 h incubation in 10 mM NO_2_^−^ in the light. (**d**) N_2_O emission rates after adding 40 µM DEA-NONOate to G1 cultures in light and dark conditions. The initial rates (100%) correspond to 0.66 ppm/h (light) and 4.42 ppm/h (dark). Each data line in (**a**) represents an average of three biological replicates, and the colored area corresponds to ±SD. Error bars represent ±SD, *n* ≥ 3. Student’s *t* test was performed. **** *p* ≤ 0.0001.

The enzymes responsible for NO reduction to N_2_O are located in the chloroplast (FLV) [27] and mitochondria (CYP55) [42] in *Chlamydomonas*. However, the NO sources that feed these reactions are not well understood. In plants and algae, the cytosolic NR seems to be the main enzyme involved in NO synthesis from NO_2_^−^ [30,31,43], although other pathways for NO synthesis have been proposed in chloroplasts [44] and mitochondria [26]. Here, we set out to elucidate the possible role of the NR–ARC complex as a NO source for the synthesis of N_2_O. First, N_2_O emissions were compared in the *nii1* mutants G1 (NR^−^) and M4 (NR^+^) (Figure 3a). The lack of NR led to a dramatic reduction in N_2_O accumulation after 24 h in both light (31 ppm) and dark (77 ppm) conditions, while the M4 strain behaved similarly to the M3 mutant, reaching 904 ppm after 24 h in the light and 395 ppm in the dark (Figure 3a,b).

Secondly, to study the potential role of ARC in N_2_O emission, we transferred the *arc* mutation to the M3 background by genetic crossing. This new strain (M3*arc*) showed a significant reduction in N_2_O accumulation after 24 h in both light (~144 ppm) and dark (~69 ppm) conditions (Figure 3a,b), suggesting that the NR–ARC complex is responsible for the synthesis of most of the NO that sustains N_2_O production. To confirm this idea, NO levels were measured in these four strains (M3, M3*arc*, M4, and G1) using the DAF-FM fluorescent probe in cells incubated for 24 h in 10 mM NO_2_^−^ under illumination (Figure 3c). G1 and M3arc strains exhibited a pronounced reduction in fluorescence (50% and 30%, respectively) compared to their corresponding strain of reference, M4, and M3. Our results suggest that NR–ARC is the main player in NO synthesis to feed NO reductases, but also that other NR–ARC-independent pathways should be considered.

If NR is required for N_2_O production as a key NO supplier, then the exogenous addition of NO should enhance N_2_O production in the NR-lacking G1 strain. To test this hypothesis, G1 cells were incubated for 20 h with 10 mM NO_2_^−^ in either light or dark conditions and then were exposed to NO donor (40 µM DEA-NONOate). In both conditions, an immediate burst of N_2_O emission was observed. Before NO donor addition, N_2_O was produced with a rate of 0.66 ppm/h and 4.42 ppm/h in light and dark, respectively; after NO donor supplementation, the rate increased up to 131 ppm/h in light and 150 ppm/h in the dark (Figure 3d). These results suggest that the low N_2_O emissions observed in the G1 strain are due to a limitation in NO synthesis.

### 2.3. Nitrite Impacts CO_2_ Emissions through a NR-Dependent Process in the nii1 Mutants

NO is a signal molecule that inhibits a wide variety of processes in *Chlamydomonas*, including photosynthesis [45] and mitochondrial respiration [46]. Thus, taking advantage of the CRDS analyzer’s functionality to quantify CO_2_, we studied CO_2_ evolution to understand how NO accumulation, and indirectly N_2_O emissions, might impact central metabolism in the *nii1* mutants. Under mixotrophic conditions, CO_2_ emissions are mainly a result of the flux balance between CO_2_ fixation (photosynthesis and Calvin–Benson–Bassham cycle) and CO_2_ release by the Tricarboxylic Acid Cycle (TCA) that is fed with acetate as an exogenous carbon source, although CO_2_ emissions can also be impacted by other processes such as carbon mobilization from storage compounds (i.e., starch and lipids) and, to a lesser extent, photorespiration [47,48,49] Therefore, we assayed how the different *nii1* mutants were affected in CO_2_ evolution.

**Figure 4:**
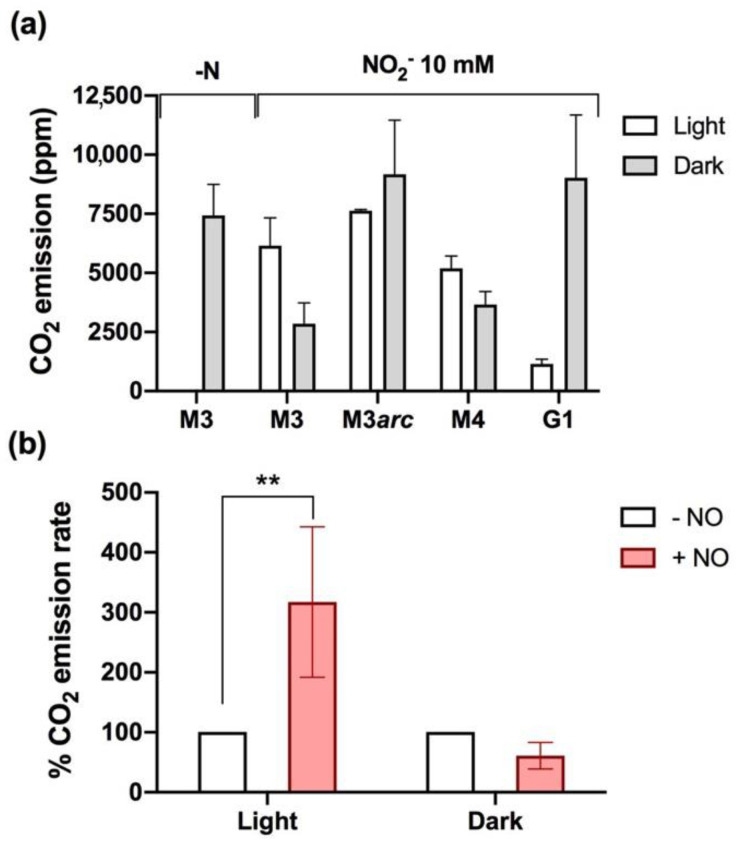
NR impacts CO_2_ evolution in the *nii1* mutants. (**a**) CO_2_ emission by the *nii1* mutants under light and dark incubation in 10 mM NO_2_^−^ and N-free media after 24 h. (**b**) CO_2_ emission rates after adding 40 µM DEA-NONOate to G1 cultures in light and dark conditions. The initial rates (100%) correspond to 44.28 ppm/h and 78.21 ppm/h in light and dark, respectively. Error bars represent ±SD, *n* ≥ 3. Student’s *t* test was performed. ** *p* ≤ 0.001.

Total CO_2_ accumulation in the headspace of the cultures was monitored after 24 h of induction in the presence of 10 mM NO_2_^−^ in light and dark conditions. In the dark, when cells cannot fix carbon, CO_2_ emissions were higher in G1 (9024 ppm) than in the M4 and M3 strains (3658 ppm and 2852 ppm) (Figure 4a and Supplementary Table S1). The same experiment, carried out under illumination, showed the opposite effect: lower CO_2_ emission in the G1 mutant (1153 ppm) than in the M4 and M3 strains (5188 ppm and 6151 ppm). Similar results were obtained for M3arc and M3 strains in the dark (M3arc accumulated more CO_2_, 9169 ppm, than M3, 2852 ppm) but not in the light, where they showed almost identical CO_2_ accumulation (Figure 4a). We suggest that this different phenotype in the light might be a consequence of the slightly higher NO levels observed in M3arc compared to G1 (Figure 3c), as CO_2_ emission patterns in light and darkness seem to be affected by NO. To test this hypothesis, the G1 cultures were supplied with a NO donor in dark and light conditions after 20 h induction in 10 mM NO_2_^−^. The NO addition led to a three-fold increase in the CO_2_ emission rate in the light but not in the dark, where only a slight reduction was observed (Figure 4b). To further confirm whether NO reduces CO_2_ emission in the dark, the M3 strain was treated with NO donor in N-free medium in the dark, and after a short incubation time (75 min) (Supplementary Figure S1). Before NO donor addition, the CO_2_ emission rate was 242 ppm/h, but after NO donor addition, the CO_2_ emission rate decreased to 88 ppm/h. Accordingly, the N_2_O emission rate increased from 0 to 8 ppm/h (Supplementary Figure S1).

CO_2_ emission was also studied in M3 cells under N deprivation and different NO_2_^−^ concentrations in the light (Supplementary Figure S2). In N-free medium, the atmospheric CO_2_ was consumed, and almost no emission was detected after 24 h. However, CO_2_ was released in the presence of NO_2_^−^ in a concentration-dependent manner (4718 ppm and 6152 ppm in 0.1 mM and 10 mM NO_2_^−^, respectively). These data highlight the regulatory role of NO_2_^−^-derived NO in CO_2_ emission levels (see Discussion Section).

### 2.4. N_2_O and CO_2_ Emissions in the NO_3_^−^/NO_2_^−^ Assimilation Wild Type Strain 6145c and the nit1nit2 Mutant CMJ030

To better understand how the NO_3_^−^/NO_2_^−^ assimilation pathway impacts N_2_O and CO_2_ emissions, we studied the accumulation of these gases in sealed cultures of the WT strain (6145c) and CMJ030, a mutant that cannot assimilate NO_3_^−^ and exhibits a limited growth on NO_2_^−^. By genetic crossing, we demonstrated that CMJ030 is a *nit1nit2* mutant (see Supplementary Figure S3) that lacks NR activity and also NIT2, which is the key transcriptional factor involved in the regulation of the NO_3_^−^/NO_2_^−^ assimilation pathway [36,40].

**Figure 5:**
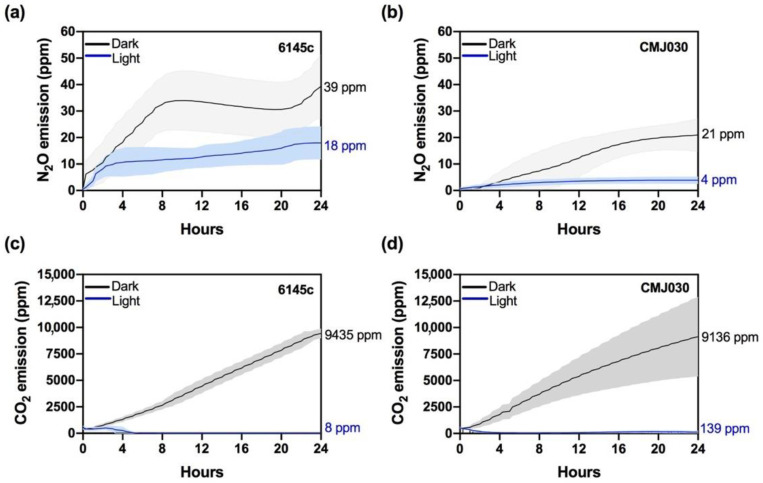
N_2_O and CO_2_ emissions in the wild-type strain 6145c and the *nit1nit*2 mutant CMJ030. NH_4_^+^-grown cells were washed and transferred to 10 mM NO_2_^−^-containing media in sealed bottles in the light (blue line) or the dark (black line). N_2_O emissions (**a**,**b**) and CO_2_ (**c**,**d**) were quantified during 24 h. Each data line represents an average of three biological replicates, and the colored area corresponds to ±SD.

Both 6145c and CMJ030 strains accumulated much less N_2_O than the M3 and M4 mutants; N_2_O emission reached 18 ppm in 6145c and 4 ppm in CMJ030 after 24 h in the light (Figure 5a,b). After normalization using chlorophyll concentration (as 6145c cultures double their chlorophyll content after 24 h in NO_2_^−^), N_2_O production in 6145c was two-fold higher than in CMJ030 (Supplementary Table S1). In the dark (where no growth was observed), normalized emission increased ~five-fold (Supplementary Table S1), showing characteristic kinetics with two phases of production separated by another phase in which N_2_O was not accumulated (Figure 5a,b). The lower N_2_O emissions observed in the *nit1nit2* mutant further support that the NO_3_^−^/NO_2_^−^ assimilation pathway impacts N_2_O synthesis in *Chlamydomonas*.

The low N_2_O production detected in these strains seems to point out that NO is not highly accumulated. Consequently, both strains exhibited high CO_2_ emissions in the dark and low CO_2_ levels in light (Figure 5c,d, and Supplementary Table S1), suggesting that 10 mM NO_2_^−^ is not enough to alter CO_2_ evolution under our experimental conditions.

## 3. Discussion

Plants and algae can produce the potent greenhouse gas N_2_O, which can be emitted at significant amounts into the atmosphere as a result of high inputs of NO_3_^−^/NO_2_^−^ [16,17,22]. Despite its potentially high environmental and ecological impact, the molecular mechanisms involved in N_2_O production by photosynthetic organisms remain largely unknown, and genetic evidence supporting N_2_O emissions has been only recently described in the model organism *Chlamydomonas reinhardtii* [26,27]. Recent works have documented the existence of two NO reductases, FLVs and CYP55, able to produce N_2_O when the alga is supplied with NO. Most of these experiments were performed in a *Chlamydomonas nit1nit2* genetic background and demonstrated that N_2_O production mostly relies on FLVs in the light and on CYP55 in the dark. Another approach by Plouviez and collaborators studied the N_2_O production from NO_2_^−^ by *Chlamydomonas* strains with different genetic backgrounds for NO_3_^−^ assimilation. Their results showed that N_2_O production by the WT strain, able to assimilate NO_3_^−^, occurs from NO_2_^−^ and mainly in the dark linked to CYP55. This result was supported later by Burlacot and collaborators showing that NO uptake and N_2_O production in the dark were much higher when WT cells were grown with NO_3_^−^ as the sole nitrogen source and reflecting the regulation of CYP55 by NO_3_^−^ metabolism. Different processes have been proposed to synthesize NO from NO_2_^−^, the intermediary step in N_2_O production [17,35,44]. Plouviez et al., 2017 [26] suggest two phases in the *Chlamydomonas* N_2_O emissions by WT in the dark, an early one involving NR (3.5 h) and a late phase involving the mitochondrial COX (24 h). Here, we present and discuss new data on the NO_2_^−^-to-N_2_O denitrification process in *Chlamydomonas nii1* mutants and how CO_2_ emissions are affected in these strains.

**When NO_3_^−^/NO_2_^−^ assimilation is interrupted because of the absence of NiR activity**, two main conclusions are considered: (1) NR and ARC (NOFNiR) have a vast impact on N_2_O emissions, and (2) this NR-dependent N_2_O emission is significantly higher (4.5-fold) in the light than in the dark, a result in accord with Plouviez et al., 2017. Our results highlight that the NO synthesized by the cytosolic NR/ARC complex can diffuse to other organelles such as mitochondria and chloroplast, and this NO seems to regulate processes involved in CO_2_ emissions (later discussed). Despite the importance of NR as the main NO source in the *nii1* mutants, the remaining NO and N_2_O levels observed in G1 cultures point out alternative NO synthesis pathways such as that involving COX, as previously reported [26].

**When NO_3_^−^/NO_2_^−^ assimilation is totally functional**, N_2_O emissions are tremendously diminished. This result reveals that N_2_O emissions in *Chlamydomonas* seem to be mainly restricted to conditions in which NO_3_^−^/NO_2_^−^ cannot be properly assimilated and used for growth. This might support why the WT strain emits more N_2_O in the dark, as cells need to acclimate to this condition and NO_3_^−^/NO_2_^−^ assimilation is less efficient. According to this, we could expect high N_2_O emission in NO_3_^−^/NO_2_^−^-rich environments depleted of other nutrients. Therefore, growth limitation in the presence of high NO_3_^−^/NO_2_^−^ concentrations should lead to high N_2_O synthesis rates. Finally, the two phases of N_2_O emission observed in dark-incubated WT cells could be attributed to NO generated by NR (first phase) and mitochondrial COX (second phase), as previously reported [17].

**When NO_3_^−^/NO_2_^−^ assimilation is impaired (*nit1nit2* mutant)**, N_2_O emissions are lower than in the WT. In this genetic background, neither NR nor the regulatory NIT2 proteins are functional, and NO_2_^−^ assimilation is slow, allowing a limited growth in this N source [50]. This residual NO_2_^−^ assimilation is enough to avoid NO_2_^−^ dissimilation to N_2_O. In addition, NIT2 also controls other steps in NO_3_^−^ assimilation, including NO_3_^−^/NO_2_^−^ transporters [36,40] and NO metabolism-related proteins such as AOX1 [51], THB1 and THB2 [38,39], and probably CYP55, which increases in response to NO_3_^−^ [29]. Moreover, a putative NO_3_^−^-dependent regulation of the N_2_O production, mediated by NIT2, is also supported by the significant increase in the N_2_O emission rate observed in M3 cells incubated in NO_3_^−^ compared to those incubated in NO_2_^−^ (Figure 2c).

CO_2_ emissions are closely related to NO_2_^−^-dependent N_2_O emissions. Our results show a relationship between N_2_O and CO_2_ emissions that will require further investigation to understand the metabolic adaptations of *Chlamydomonas* to heterotrophic and mixotrophic conditions in the presence of NO_3_^−^ or NO_2_^−^. In both conditions, acetate is the main carbon source, but it is essential only in the dark to feed the TCA cycle and provide energy to the cells, releasing CO_2_ [47,52,53].

This study shows that low N_2_O emissions correlate with high CO_2_ release in the dark and vice versa; high N_2_O emissions correlate with less CO_2_ release. The link between N_2_O and CO_2_ emissions appears to be the NO signal molecule, produced mainly by the NR/ARC complex in the *nii1* mutants. NO could inhibit acetate metabolism and CO_2_ release, also supported by the slight inhibition of the CO_2_ emission rate by NO donor.

In the light, we found the opposite correlation: low N_2_O accumulation, due to low NO synthesis, leads to a reduced CO_2_ emission and vice versa. Under illumination, NO inhibits photosynthesis [45], reducing CO_2_ fixation. In fact, NO supply increased by three-fold the CO_2_ emissions in the light, suggesting that CO_2_ fixation is very sensitive to NO. Thus, CO_2_ fixation would be more active in those strains/conditions in which low NO is synthesized (low N_2_O emitted) and, therefore, lower CO_2_ levels would be accumulated. The role of NO as a photosynthesis inhibitor has been described in plants and algae and has been considered a mechanism to avoid photo-damage in algae under nutrient deprivation [45,54,55]. Nitrogen- [56] or sulfur-starved [57] *Chlamydomonas* cells accumulate NO, which causes the degradation of the cytochrome b_6_*f* complex and Rubisco by the FtsH and Clp proteases. More recently, transcriptomic analyses reported the molecular mechanisms underlying the acclimation of *Chlamydomonas* after NO supply [45]. Among the regulated process, NO decreases photosynthesis, respiration, N availability, and induces NO scavenging (THB1, THB2, FLVB, and CYP55).

The contributions of plants and algae to the N_2_O atmospheric budget are not being considered by the expert panels, even when increasing reports support their participation in this process, and the high input of nitrogen fertilizers is the primary cause [8,17,22]. Our data shed light on the mechanisms involved in the N_2_O synthesis and highlight the *nii1* mutants as good models to study the molecular bases of the N_2_O emission in photosynthetic organisms. Moreover, the NR role on N_2_O emission raises an important link between NO_3_^−^ assimilation and dissimilation, making of this enzyme a good candidate for future studies in order to acquire a better understanding on those environmental conditions that promote NO_3_^−^ dissimilation over assimilation.

## 4. Materials and Methods

### 4.1. Strains and Growth Conditions

The strains used in this study are listed in Supplementary Table S1. G1 strain is a deletion mutant affected at the *NIT1 locus* and lacking nitrite reductase (NiR), nitrate reductase (NR) and the high-affinity NO_3_^−^/NO_2_^−^ transporters (∆ (*NII1*, *NIA1*, *NRT2.2*, *NRT2.1*, *NAR2*)). By genetic crosses, *NIA1* (the gene encoding NR) was transferred to G1 to generate the M4 strain (∆ (*NII1*, *NIA1*, *NRT2.2*, *NRT2.1*, *NAR2*):*NIA1*). Similarly, *NIA1* plus the gene encoding the NO_3_^−^/NO_2_^−^ transporter *NRT2.1* were added to obtain the M3 strain (∆ (*NII1*, *NIT1*, *NRT2.2*, *NRT2.1*, *NAR2*):*NIT1*, :(*NRT2.1*, *NAR2*)) [41]. Strain 6145c is a WT strain for NO_3_^−^ assimilation and CMJ030 is a *nit1nit2* mutant. Finally, the M3*arc* strain (∆ (*NII1*, *NIT1*, *NRT2.2*, *NRT2.1*, *NAR2*):*NIT1*, :(*NRT2.1, NAR2*), *arc*) was obtained in this work by crossing M3 (mt^+^) and LMJ.RY0402.255418 (mt^−^), where LMJ.RY0402.255418 is an insertional mutant where the *ARC* gene was interrupted with the paramomycin cassette [58] (obtained from the *Chlamydomonas* Library Project (CLiP), https://www.chlamylibrary.org).

All the cell cultures were performed in TAP medium (Tris, Acetate, Phosphate) [59] in a chamber (AlgaeTron AG 230, Photon System Instruments, Drásov, Czech Republic) at 25 °C, with continuous agitation (120 rpm) and illumination (light intensity 130 µmol photons·s^−1^·m^−2^). When indicated, cell cultures were transferred to dark in the same chamber.

Cells were grown in TAP medium with NH_4_^+^ as a nitrogen source (8 mM NH_4_Cl) (pre-cultures). At the exponential phase of the culture, cells were harvested by centrifugation (2 min at 3000× *g*), washed twice with nitrogen-free TAP and transferred to new media containing the indicated nitrogen sources. The initial chlorophyll concentration was adjusted to 9–10 μg mL^−1^.

For unsealed flask, Erlenmeyer flasks covered with foil paper were used. The same flasks were hermetically sealed with screw caps (sealed flasks), and a syringe was used to collect samples from the culture.

### 4.2. Chlorophyll, NO_2_^−^, and Cell Counting Measurements

Samples of 1 mL were centrifuged at 15,000× *g* for 5 min, and the supernatant (cell-free medium) and the pellet were separately frozen at −20 °C. NO_2_^−^ was quantified in the cell-free medium using the Griess reagents according to Snell and Snell (1949) [60]. For chlorophyll concentration, the pellet was resuspended in 1 mL ethanol and incubated for 3 min, at room temperature. Afterwards, the samples were centrifuged and the chlorophyll concentration in the supernatant was quantified as previously described [61]. Cell quantification of liquid cultures was determined using the Sysmex Microcellcounter F-500 cell counter.

### 4.3. NO Measurements

Cells cultures (25 mL) were induced in media with 10 mM NO_2_^−^ during 24 h. Then, 2 µM of DAF–FM (4,5-Diaminofluorescein) was added and incubated for 1h. An amount of 200 µL of the culture was used for NO quantification in a fluorescence spectrophotometer (Varioskan Lux, Thermo scientific, Waltham, MA USA) using OptiPlate Black Opaque 96-well Microplate (PerkinElmer, Waltham, MA USA). The excitation and emission wavelengths for the NO indicator were 485 and 515 nm, respectively. Data are represented as arbitrary fluorescence units.

### 4.4. Determination of N_2_O and CO_2_ Emissions

N_2_O and CO_2_ were simultaneously quantified by using a Cavity Ring-Down Spectroscopy (CRDS) analyzer (PICARRO G2508). For this purpose, we used 1 L bottles (DURAN^TM^) that were hermetically sealed with screw caps (GL 45 with 2 or 3 connectors) both from DWK Life Sciences (Mainz, Germany). The bottles were set with 250 mL liquid culture medium and 750 mL headspace (gas phase). The CRDS analyzer was connected to the bottle through a combined inlet and outlet Teflon tubes (2.5 m in length). The outlet tube extracted the sample from the headspace of the bottle (0.3 L/min), and the inlet tube returned the sample into the gas phase of the cultures, passing the air through a 0.22 µm PVDF filter (Dualex^TM^-Plus; Merck, Darmstadt, Germany) to avoid culture contamination.

### 4.5. Genetic Crosses

Genetic crosses were performed according to [62] and the random spore plating method. Then, 100 segregants were analyzed, and several of them were chosen for further experiments.

### 4.6. Chemicals and Statistical Analysis

DEA-NONOate [2-(N,N-diethylamino)-diazenolate 2-oxide sodium salt] (D-184) and DAF–FM (4,5-Diaminofluorescein) (D224-1MG) were purchased from Merck (Darmstadt, Germany). For statistical analysis (Student’s *t* test), PRISM software v8.4.3 (GraphPad Software, LLC, San Diego, CA, USA) was used.

## Data Availability

All data required to evaluate the conclusions of this paper are included in the main text or the Supplementary Materials.

## Supplementary Materials

The supporting information can be downloaded at: https://www.mdpi.com/article/10.3390/ijms23169412/s1.

## References

[1] Kuypers M.M.M., Marchant H.K., Kartal B. (2018). The microbial nitrogen-cycling network. Nat. Rev. Microbiol..

[2] Ravishankara A.R., Daniel J.S., Portmann R.W. (2009). Nitrous oxide (N_2_O): The dominant ozone-depleting substance emitted in the 21st century. Science.

[3] Crutzen P.J., Mosier A.R., Smith K.A., Winiwarter W. (2008). N_2_O release from agro-biofuel production negates global warming reduction by replacing fossil fuels. Atmos. Chem. Phys..

[4] Davidson E.A. (2009). The contribution of manure and fertilizer nitrogen to atmospheric nitrous oxide since 1860. Nat. Geosci..

[5] Prather M.J., Hsu J., Deluca N.M., Jackman C.H., Oman L.D., Douglass A.R., Fleming E.L., Strahan S.E., Steenrod S.D., Søvde O.A. (2015). Measuring and modeling the lifetime of nitrous oxide including its variability. J. Geophys. Res. Atmos..

[6] Basso L., Crotwell A., Dolman H., Gatti L., Gerbig C., Griffith D., Hall B., Jordan A., Krummel P., Leuenberger M. (2021). The state of greenhouse gases in the atmosphere based on global observations through 2020. WMO Greenh. Gas Bull..

[7] Masson-Delmotte V., Zhai P., Pirani A., Connors S.L., Péan C., Berger S., Caud N., Chen Y., Goldfarb L., Gomis M.I. (2021). IPCC, 2021: Summary for Policymakers. Climate Change 2021: The Physical Science Basis. Contribution of Working Group I to the Sixth Assessment Report of the Intergovernmental Panel on Climate Change.

[8] Tian H., Xu R., Canadell J.G., Thompson R.L., Winiwarter W., Suntharalingam P., Davidson E.A., Ciais P., Jackson R.B., Janssens-Maenhout G. (2020). A comprehensive quantification of global nitrous oxide sources and sinks. Nature.

[9] Seitzinger S.P., Kroeze C. (1998). Global distribution of nitrous oxide production and N inputs in freshwater and coastal marine ecosystems. Glob. Biogeochem. Cycles.

[10] Liu L., Greaver T.L. (2009). A review of nitrogen enrichment effects on three biogenic GHGs: The CO_2_ sink may be largely offset by stimulated N_2_O and CH_4_ emission. Ecol. Lett..

[11] Richardson D., Felgate H., Watmough N., Thomson A., Baggs E. (2009). Mitigating release of the potent greenhouse gas N_2_O from the nitrogen cycle—Could enzymic regulation hold the key?. Trends Biotechnol..

[12] Maeda K., Spor A., Edel-Hermann V., Heraud C., Breuil M.C., Bizouard F., Toyoda S., Yoshida N., Steinberg C., Philippot L. (2015). N_2_O production, a widespread trait in fungi. Sci. Rep..

[13] Shoun H., Fushinobu S., Jiang L., Kim S.W., Wakagi T. (2012). Fungal denitrification and nitric oxide reductase cytochrome P450nor. Philos. Trans. R. Soc. B Biol. Sci..

[14] Higgins S.A., Welsh A., Orellana L.H., Konstantinidis K.T., Chee-Sanford J.C., Sanford R.A., Schadt C.W. (2016). Detection and diversity of fungal nitric oxide reductase genes (P450nor) in agricultural soils. Appl. Environ. Microbiol..

[15] Hahn J., Junge C. (1977). Atmospheric nitrous oxide: A critical review. Z. Nat. A.

[16] Weathers P.J. (1984). N_2_O evolution by green algae. Appl. Environ. Microbiol..

[17] Plouviez M., Shilton A., Packer M.A., Guieysse B. (2019). Nitrous oxide emissions from microalgae: Potential pathways and significance. J. Appl. Phycol..

[18] Dean J.V., Harper J.E. (1986). Nitric oxide and nitrous oxide production by soybean and winged bean during the in vivo nitrate reductase assay. Plant Physiol..

[19] Goshima N., Mukai T., Suemori M., Takahashi M., Caboche M., Morikawa H. (1999). Emission of nitrous oxide (N_2_O) from transgenic tobacco expressing antisense NiR mRNA. Plant J..

[20] Smart D.R., Bloom A.J. (2001). Wheat leaves emit nitrous oxide during nitrate assimilation. Proc. Natl. Acad. Sci. USA.

[21] Hakata M., Takahashi M., Zumft W., Sakamoto A., Morikawa H. (2003). Conversion of the nitrate nitrogen and nitrogen dioxide to nitrous oxides in plants. Acta Biotechnol..

[22] Lenhart K., Behrendt T., Greiner S., Steinkamp J., Well R., Giesemann A., Keppler F. (2019). Nitrous oxide effluxes from plants as a potentially important source to the atmosphere. New Phytol..

[23] Bartram D., Short M.D., Ebie Y., Farkaš J., Gueguen C., Peters G.M., Zanzottera N.M., Karthik M., Masuda S. (2019). Wastewater treatment and discharge. 2019 Refinement to the 2006 IPCC Guidelines for National Greenhouse Gas Inventories.

[24] Salomé P.A., Merchant S.S. (2019). A series of fortunate events: Introducing *Chlamydomonas* as a reference organism. Plant Cell.

[25] Sasso S., Stibor H., Mittag M., Grossman A.R. (2018). From molecular manipulation of domesticated *Chlamydomonas reinhardtii* to survival in nature. eLife.

[26] Plouviez M., Wheeler D., Shilton A., Packer M.A., McLenachan P.A., Sanz-Luque E., Ocaña-Calahorro F., Fernández E., Guieysse B. (2017). The biosynthesis of nitrous oxide in the green alga *Chlamydomonas reinhardtii*. Plant J..

[27] Burlacot A., Richaud P., Gosset A., Li-Beisson Y., Peltier G. (2020). Algal photosynthesis converts nitric oxide into nitrous oxide. Proc. Natl. Acad. Sci. USA.

[28] Folgosa F., Martins M.C., Teixeira M. (2018). The multidomain flavodiiron protein from *Clostridium difficile* 630 is an NADH: Oxygen oxidoreductase. Sci. Rep..

[29] Bellido-Pedraza C.M., Calatrava V., Sanz-Luque E., Tejada-Jiménez M., Llamas A., Plouviez M., Guieysse B., Fernandez E., Galvan A. (2020). *Chlamydomonas reinhardtii*, an algal model in the nitrogen cycle. Plants.

[30] Yamasaki H., Sakihama Y. (2000). Simultaneous production of nitric oxide and peroxynitrite by plant nitrate reductase: In vitro evidence for the NR-dependent formation of active nitrogen species. FEBS Lett..

[31] Rockel P., Strube F., Rockel A., Wildt J., Kaiser W.M. (2002). Regulation of nitric oxide (NO) production by plant nitrate reductase in vivo and in vitro. J. Exp. Bot..

[32] Sakihama Y., Nakamura S., Yamasaki H. (2002). Nitric oxide production mediated by nitrate reductase in the green alga *Chlamydomonas reinhardtii*: An alternative NO production pathway in photosynthetic organisms. Plant Cell Physiol..

[33] Timilsina A., Zhang C., Pandey B., Bizimana F., Dong W., Hu C. (2020). Potential pathway of nitrous oxide formation in plants. Front. Plant Sci..

[34] Campbell W.H. (2001). Structure and function of eukaryotic NAD(P)H: Nitrate reductase. Cell. Mol. Life Sci..

[35] Chamizo-Ampudia A., Sanz-Luque E., Llamas A., Galvan A., Fernandez E. (2017). Nitrate reductase regulates plant nitric oxide homeostasis. Trends Plant Sci..

[36] Fernandez E., Galvan A. (2008). Nitrate assimilation in *Chlamydomonas*. Eukaryot. Cell.

[37] Chamizo-Ampudia A., Sanz-Luque E., Llamas Á., Ocaña-Calahorro F., Mariscal V., Carreras A., Barroso J.B., Galván A., Fernández E. (2016). A dual system formed by the ARC and NR molybdoenzymes mediates nitrite-dependent NO production in *Chlamydomonas*. Plant Cell Environ..

[38] Johnson E.A., Rice S.L., Preimesberger M.R., Nye D.B., Gilevicius L., Wenke B.B., Brown J.M., Witman G.B., Lecomte J.T.J. (2014). Characterization of THB1, a *Chlamydomonas reinhardtii* truncated hemoglobin: Linkage to nitrogen metabolism and identification of lysine as the distal heme ligand. Biochemistry.

[39] Sanz-Luque E., Ocaña-Calahorro F., De Montaigu A., Chamizo-Ampudia A., Llamas Á., Galván A., Fernández E. (2015). THB1, a truncated hemoglobin, modulates nitric oxide levels and nitrate reductase activity. Plant J..

[40] Sanz-Luque E., Chamizo-Ampudia A., Llamas A., Galvan A., Fernandez E. (2015). Understanding nitrate assimilation and its regulation in microalgae. Front. Plant Sci..

[41] Navarro M.T., Guerra E., Fernandez E., Galvan A. (2000). Nitrite reductase mutants as an approach to understanding nitrate assimilation in *Chlamydomonas reinhardtii*. Plant Physiol..

[42] Gérin S., Mathy G., Blomme A., Franck F., Sluse F.E. (2010). Plasticity of the mitoproteome to nitrogen sources (nitrate and ammonium) in *Chlamydomonas reinhardtii*: The logic of Aox1 gene localization. Biochim. Biophys. Acta-Bioenerg..

[43] Yamasaki H., Sakihama S., Takahashi S. (1999). An alternative pathway for nitric oxide production in plants: New features of an old enzyme. Trends Plants Sci..

[44] Astier J., Gross I., Durner J. (2018). Nitric oxide production in plants: An update. J. Exp. Bot..

[45] Kuo E.Y.H., Lee T.M. (2021). Molecular mechanisms underlying the acclimation of *Chlamydomonas reinhardtii* against nitric oxide stress. Front. Plant Sci..

[46] Zalutskaya Z., Kochemasova L., Ermilova E. (2018). Dual positive and negative control of *Chlamydomonas* PII signal transduction protein expression by nitrate/nitrite and NO via the components of nitric oxide cycle. BMC Plant Biol..

[47] Johnson X., Alric J. (2013). Central carbon metabolism and electron transport in *Chlamydomonas reinhardtii*: Metabolic constraints for carbon partitioning between oil and starch. Eukaryot. Cell..

[48] Shi X., Bloom A. (2021). Photorespiration: The Futile Cycle?. Plants.

[49] Schnell R.A., Lefebvre P.A. (1993). Isolation of the *Chlamydomonas* regulatory gene NIT2 by transposon tagging. Genetics.

[50] Quesada A., Gómez-García I., Fernández E. (2000). Involvement of chloroplast and mitochondria redox valves in nitrate assimilation. Trends Plant Sci..

[51] Singh H., Shukla M.R., Chary K.V., Rao B.J. (2014). Acetate and bicarbonate assimilation and metabolite formation in *Chlamydomonas reinhardtii*: A ^13^C-NMR Study. PLoS ONE.

[52] Sweetlove L.J., Beard K.F.M., Nunes-Nesi A., Fernie A.R., Ratcliffe R.G. (2010). Not just a circle: Flux modes in the plant TCA cycle. Trends Plant Sci..

[53] Peltier G., Schmidt G.W. (1991). Chlororespiration: An adaptation to nitrogen deficiency in *Chlamydomonas reinhardtii*. Proc. Natl. Acad. Sci. USA.

[54] Grossman A.R. (2000). Acclimation of *Chlamydomonas reinhardtii* to its nutrient environment. Protist.

[55] Salomon E., Bar-Eyal L., Sharon S., Keren N. (2013). Balancing photosynthetic electron flow is critical for cyanobacterial acclimation to nitrogen limitation. Biochim. Biophys. Acta-Bioenerg..

[56] Wei L., Derrien B., Gautier A., Houille-Vernes L., Boulouis A., Saint-Marcoux D., Malnoë A., Rappaport F., de Vitry C., Vallon O. (2014). Nitric oxide-triggered remodeling of chloroplast bioenergetics and thylakoid proteins upon nitrogen starvation in *Chlamydomonas reinhardtii*. Plant Cell.

[57] De Mia M., Lemaire S.D., Choquet Y., Wollman F.A. (2019). Nitric oxide remodels the photosynthetic apparatus upon S-starvation in *Chlamydomonas reinhardtii*. Plant Physiol..

[58] Li X., Patena W., Fauser F., Jinkerson R.E., Saroussi S., Meyer M.T., Ivanova N., Robertson J.M., Yue R., Zhang R. (2019). A genome-wide algal mutant library and functional screen identifies genes required for eukaryotic photosynthesis. Nat. Genet..

[59] Harris E.H., Harris E. (1989). Culture and storage methods. The Chlamydomonas Sourcebook. A Comprehensive Guide to Biology and Laboratory Use.

[60] Snell F.D., Snell C.T. (1949). Colorimetric Methods of Analysis.

[61] Arnon D.I. (1949). Copper enzymes in isolated chloroplasts. Polyphenoloxidase in *Beta vulgaris*. Plant Physiol..

[62] Jiang X., Stern D. (2009). Mating and tetrad separation of *Chlamydomonas reinhardtii* for genetic analysis. J. Vis. Exp..

