# Supplementary material for: Nitrous Oxide Emissions from Nitrite Are Highly Dependent on Nitrate Reductase in the Microalga Chlamydomonas reinhardtii
Source: Int J Mol Sci. 2022 Aug 20;23(16):9412. doi: 10.3390/ijms23169412 (PMC9409008; doi:10.3390/ijms23169412)
Supplement: Supplementary file 1 [file ijms-23-09412-s001.zip › ijms-1846367-supplementary.pdf]

Supplementary Figure S1

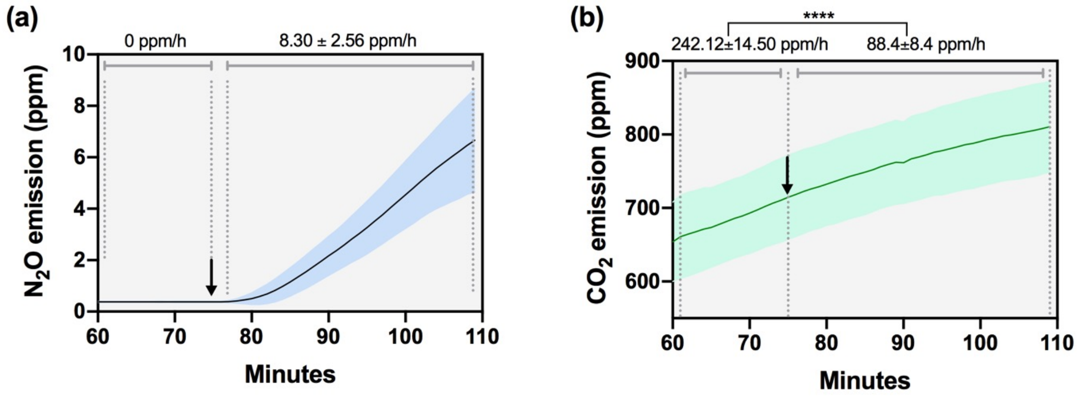

Figure S1. Effect of NO on N_2O and CO_2 emissions in N starved M3 cells in the dark. Ammonium-grown cells were washed and transferred to N-free media in the dark. After 75 min, 40 μM DEA-NONOate was added (arrow) and N_2O (**a**) and CO_2 (**b**) emissions were monitored and the rate was calculated. As shown, the N_2O emission rate was triggered and the CO_2 emission rate was slowed down by exogenous NO supplementation. Each data line represents an average of three biological replicates, and the colored area corresponds to ±SD. Student's *t* test was performed. ****P ≤ 0.0001.

Supplementary Figure S2

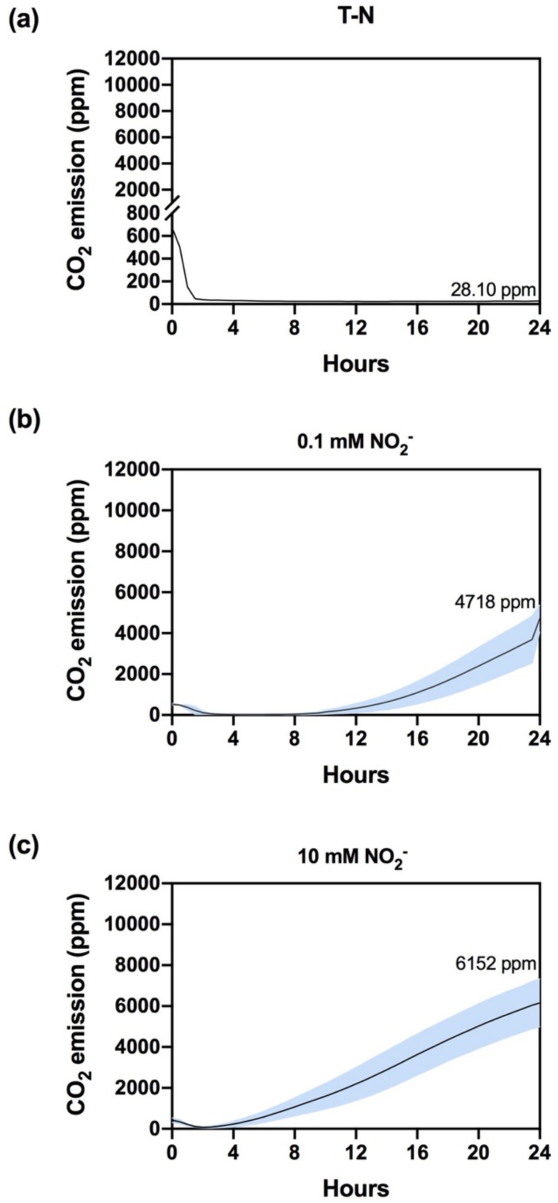

Figure S2. CO_2 emission in light-induced M3 cells depends on NO_2^- concentrations. Ammonium-grown cells were washed and transferred to the indicated medium, and CO_2 emission was monitored for 24 h in the light. As shown, in N free medium (**a**) the atmospheric CO_2 present at the beginning of the experiment (~600 ppm) was consumed and no CO_2 emission was later observed. In the presence of 0.1 mM NO_2^- (**b**), the initial CO_2 present in the headspace was also initially consumed, but CO_2 emission was later detected after 8 h. In the presence of 10 mM NO_2^- (**c**), the atmospheric CO_2 in the headspace was consumed, but CO_2 emission was detected after 3 h. Each data line represents an average of three biological replicates, and the colored area corresponds to ±SD.

Supplementary Figure S3

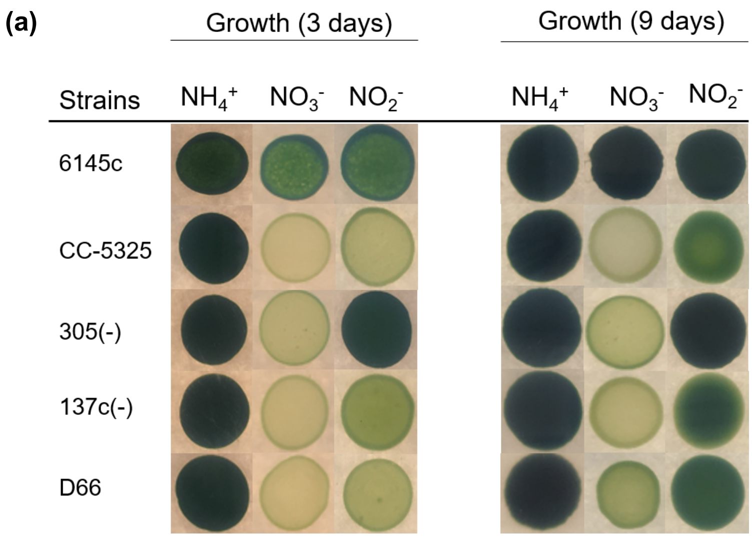

(b)

**Growth**	**NH_4^+**	**NO_3^-**	**NO_2^-**
Positive	100	30	55
Negative	0	70	45
Total	100	100	100

Figure S3. CMJ030 (CC-5325) is a *nitnit2* mutant. (**a**) growth test in NH_4^+, NO_3^- and NO_2^- of different *Chlamydomonas* strains, 6145c (wild-type, *NIT1 NIT2*), 305 (*nit1 NIT2*), D66 (*NIT1 nit2*), 137c (*nit1 nit2*) and CC-5325 (*nit1 nit2*). (**b**) Number of segregants, obtained from the genetic cross between 21gr (CC-1690) and CC-5325 strain, that grow in the indicated media.

SUPPLEMENTARY TABLE S1

Supplementary Table S1. *Chlamydomonas* strains used in this work and N_2O and CO_2 emissions in light and dark conditions

**LIGHT**									
		**N_2O emission after 24 h**		**CO_2 emission after 24 h**	**Chlorophyll (µg/ml)**		**NO_2^- (mM)**	**pH**	
**Strains / Genotype**	**Culture conditions**	**ppm**	**nmol N_2O/mgChl**	**CO_2 (ppm)**	**0 h**	**24 h**	**24 h**	**0 h**	**24 h**
**G1*** / *Δ (NII1, NIA1, NRT2.2, NRT2.1, NAR2)*	NO_2^-10 mM	30.9±1.14	414.5±30.08	1153.3±194.74	9.4±0.98	10.00±0.41	9.51±0.63	7.73±0.01	7.97±0.11
**M4*** / *Δ (NII1, NIA1, NRT2.2, NRT2.1, NAR2) ::NIA1*		904.67±145	119070.1±1338.34	5188.67±527.34	9.48±1.34	10.12±1.15	9.18±0.37	7.66±0.03	7.91±0.09
**M3*** / *Δ (NII1, NIT1, NRT2.2, NRT2.1, NAR2) ::NIT1, ::(NRT2.1, NAR2*)		864.04±95.30	14246.01±2624.68	6151.74±1174.89	8.79±2.31	8.29±1.17	9.45±1.16	7.62±0.09	7.87±0.14
**M3*arc***** */ Δ (NII1, NIT1, NRT2.2, NRT2.1, NAR2) ::NIT1, ::(NRT2.1, NAR2*), *arc*		144.44±54.39	2148.02±701.94	7626.78±60.46	9.78±1.12	8.90±0.52	9.75±0.41	7.68±0.05	7.86±0.11
**6145c** / WT		17.97±6.08	112.40±37.32	8.11±4.75	10.58±0.19	21.37±0.23	7.93±2.57	7.54±0.05	7.83±0.06
**CMJO30***** / *nit1nit2*		3.86±1.22	56.71±18.90	138.98±86.56	9.58±1.0	9.15±0.24	9.71±0.57	7.5±0.05	7.5±0.06
**M3** / *Δ (NII1, NIT1, NRT2.2, NRT2.1, NAR2) ::NIT1, ::(NRT2.1, NAR2*)	T-N	0,46	6,24	28,1	9,45	9,87	-	7,53	7,59
W/O cells		0.40±0.01	-	431.70±9.21	-	-	9.96±0.47	7.55±0.04	7.49±0.07
**DARK**									
**G1** / *Δ (NII1, NIT1, NRT2.2, NRT2.1, NAR2)*	NO_2^- 10 mM	77.03±34.41	1093.27±519.96	9024.34±2660.84	9.17±0.20	9.51±0.37	9.31±0.14	7.52±0.12	7.88±0.08
**M4** / *Δ (NII1, NIA1, NRT2.2, NRT2.1, NAR2) ::NIA1*		395.67±120.33	6424.24±1799.50	3658.55±553.67	8.59±0.65	8.21±0.23	9.54±0.70	7.66±0.01	7.64±0.01
**M3** / *Δ (NII1, NIT1, NRT2.2, NRT2.1, NAR2) ::NIT1, ::(NRT2.1, NAR2*)		269.96±35.13	4676.93±785.70	2852.27±883.54	8.67±1.41	7.78±0.53	9.40±0.43	7.62±0.18	7.73±0.00
**M3*arc*** */ Δ (NII1, NIT1, NRT2.2, NRT2.1, NAR2) ::NIT1, ::(NRT2.1, NAR2*), *arc*		75.27±23.18	1064.76±321.18	9169.14±2292.31	9.79±0.25	9.45±0.20	9.79±0.24	7.58±0.11	7.54±0.13
**6145c** / WT		39.34±11.85	590.44±218.46	9435.38±406.73	8.84±0.78	9.09±0.65	8.93±0.62	7.77±0.07	8.06±0.27
**CMJO30** / *nit1nit2*		20.99±6.16	315.54±96.86	9136.33±3712.46	9.08±0.08	8.93±0.15	9.03±0.58	7.68±0.07	8.1±0.02
**M3** / *Δ (NII1, NIT1, NRT2.2, NRT2.1, NAR2) ::NIT1, ::(NRT2.1, NAR2*)	T-N	0.43±0.01	6,29±0.20	7435.03±1306.39	9.10±0.08	9.15±0.13	-	7.58±0.07	7.68±0.02

The data are corresponding to that after 24 h incubation in the indicated media and represent mean ± SD from three biological replicates. *Δ* indicates deletion, :: means insertion and the genes mutated either by point mutations of by insertional mutagenesis are indicates using lowercase and italics. * *nii1* mutants previously isolated (Navarro et al. 2000). ** *nii1* mutant isolated in this work. *** purchased from the *Chlamydomonas* library (CLiP) (https://www.chlamylibrary Other details, see Material and Methods.
